# The burden of hypertensive heart disease in China and G20 countries: analysis from 1990 to 2021 and 30-year projections for China

**DOI:** 10.3389/fcvm.2026.1728877

**Published:** 2026-02-26

**Authors:** Yingzi Zhang, Zhaoyu Peng, Yongjin Chen, Xinzheng Tang

**Affiliations:** 1 Shenzhen Hospital (Futian) of Guangzhou University of Chinese Medicine, Shenzhen, China; 2 The Sixth Clinical Medical College, Guangzhou University of Chinese Medicine, Shenzhen, China

**Keywords:** disability-adjusted life years, hypertensive heart disease, joinpoint regression, mortality, prevalence

## Abstract

**Background:**

Hypertensive heart disease (HHD) poses a major global public health challenge. This study provides updated estimates of the HHD burden in China and G20 countries from 1990 to 2021 and projects China's trends to 2051.

**Methods:**

Using data from the Global Burden of Disease 2021 study, we analyzed HHD prevalence, mortality, DALYs, and age-standardized rates (ASPR, ASDR) for China and G20 countries. Joinpoint regression was used to calculate annual percentage change (APC) and average annual percentage change (AAPC). An ARIMA model projected China's incidence for 2021–2051.

**Results:**

In 2021, China reported 39.12 million HHD cases, 32.81 million deaths, and 55.89 million DALYs, with ASPR, ASDR, and DALY rates of 192.47, 18.85, and 292.54 per 100,000, respectively. G20 countries had 90.81 million cases, 89.67 million deaths, and 160.18 million DALYs, with corresponding rates of 139.64, 14.06, and 248.75 per 100,000. From 1990 to 2021, AAPCs for ASPR and ASDR showed no significant changes in China or G20 countries. China's rates consistently exceeded G20 averages, with no marked gender differences. Projections indicate a rising ASPR but slightly declining ASDR in China by 2051.

**Conclusion:**

Both China and the G20 group exhibit a pronounced upward trend in HHD prevalence and mortality, likely attributable to the backdrop of population aging. Conversely, China's declining prevalence and mortality rates may benefit from advances in medical technology. Furthermore, China's ASPR is projected to increase annually over the next 30 years. These findings underscore the imperative to strengthen prevention, control, and treatment strategies, providing crucial evidence for healthcare planning and practice.

## 1 Introduction

Hypertensive heart disease (HHD) is a cardiac dysfunction caused by hypertension that has gradually become a major global health issue. Initially considered an adaptive response, HHD progression not only leads to structural and functional cardiac alterations but is also closely associated with complications such as heart failure, atrial fibrillation, and coronary artery disease. HHD has become the second leading cause of heart failure (HF) (1–4). According to the World Health Organization (WHO), hypertension is a prevalent chronic disease, yet global blood pressure control rates remain alarmingly low at only 32.5% among treated individuals, affecting the health of billions worldwide (3). Concurrently, hypertension prevalence increases significantly with age, affecting approximately 65% of adults aged 60–79 and about 80% of those aged 80 and above (5). As global aging accelerates, the proportion of elderly populations is rising significantly. In 1990, individuals aged 60 and above constituted 9.2% of the global population; by 2013, this proportion had increased to 11.7%, and it is projected to reach 21.1% by 2051 (6). As the world's second most populous nation, China faces a substantial hypertension burden. In 2021, it reported a staggering 245 million cases of hypertension, with a prevalence rate as high as 15%. However, less than 15% of affected individuals received adequate treatment (7, 8). Consequently, hypertension-induced heart disease (HHD) has become a significant public health concern among the elderly, particularly noteworthy against the backdrop of extended human life expectancy (9).

The dramatic shifts in dietary patterns and lifestyles during periods of rapid economic growth represent another major contributor to HHD. Studies indicate that elderly hypertensive patients with altered dietary habits, sedentary lifestyles, elevated BMI, and dyslipidemia face substantially higher risks of heart disease (10–12). Current diets predominantly consist of high-calorie, energy-dense foods, while lifestyles increasingly feature sedentary habits, reduced engagement in traditional labor-intensive activities, and declining physical activity levels. This has led to rising rates of overweight and obesity. Obesity and its associated risks are the primary drivers of the increasing incidence of hypertension, causing more deaths than any other cardiovascular risk factor (2). Despite significant improvements in the treatment and management of arterial hypertension over the past decades, the prevalence of HHD and its associated risk of heart failure continue to rise. This underscores that HHD remains a critical public health issue warranting high attention and priority. However, to date, the vast majority of research on HHD has focused on its pathogenesis and therapeutic approaches. Epidemiological studies can reveal the burden of HHD on human health from a macro perspective, providing evidence for strengthening public health at the national level, prudently allocating medical resources, and formulating health strategies.

Therefore, as the G20 is an international economic cooperation forum comprising both developed and developing nations, whose member countries account for approximately 85% of global GDP（Gross Domestic Product) and two-thirds of the world's population, it represents a group of nations that dominate the global economic and political landscape (13). This study aims to systematically evaluate the disease burden of HHD and its temporal trends in China and the G20 as a whole (including China) from 1990 to 2021. It analyzes the characteristics of disease burden within this significant group and forecasts the incidence and mortality trends of HHD in China over the next 30 years. Currently, comparative studies examining the HHD disease burden between China and other nations remain scarce. This lack of comparative research hinders China's ability to identify shortcomings in its health policies and practices while limiting its capacity to draw lessons from effective strategies and experiences of other countries. By comparing China with G20 nations, this study not only provides an opportunity to learn from successful strategies implemented by other G20 countries but also offers valuable insights for nations at similar stages of economic development (14).

## 2 Materials and methods

### 2.1 Definitions

The GBD 2021 defines HHD (ICD-11 code: BA01) as heart disease resulting from the direct or indirect effects of hypertension. It should be noted that diagnostic thresholds for hypertension may vary across countries or regions and over time. The Global Burden of Disease (GBD) study employs standardized modeling methods to provide comparable estimates across time and geography; however, residual diagnostic differences may constitute a limitation. The age-standardized prevalence rate (ASPR) represents the age-standardized number of current cases (including new and past cases) per 100,000 population, adjusted based on a standard population age structure. Age-Standardized Death Rate (ASDR) Represents the age-standardized number of deaths from a specific disease per 100,000 population. Disability-adjusted life years (DALYs) measure the loss of healthy life years due to disease, disability, or premature death. The age-standardized DALY rate represents the total age-standardized healthy life years lost per 100,000 population due to a specific disease or injury. DALYs are calculated by summing YLLs and YLDs (DALYs = YLLs + YLDs). Here, disability weight (DW) represents the severity of health impairment or non-fatal disability. Years of life lost (YLDs) are calculated by multiplying the total number of cases by the duration of impairment or survival, then by DW. Years of life lost (YLLs) are determined by multiplying the number of deaths by projected life expectancy based on age, sex, location, and year (15). The Annual Percentage Change (APC) indicates the average annual rate of change in a disease burden indicator over a specific period, reflecting short-term trends. The Average Annual Percentage Change (AAPC) represents the overall average rate of change in a disease burden indicator throughout the entire study period. The uncertainty interval indicates the probability that the true value lies within this range at the 95% confidence level.

### 2.2 Data sources

HHD disease burden data for China and G20 countries (including all G20 members such as Canada, France, and the United States) from 1990 to 2021 were obtained from the GBD 2021 Public Database (https://vizhub.healthdata.org/gbd-results). For our study, we accessed prevalence, mortality, and DALY estimates related to HHD. The GBD 2021 study employed the latest epidemiological data and improved standardization methods, utilizing 100,983 data sources to comprehensively assess health loss from 369 diseases, injuries, and 88 risk factors across 204 countries and regions (16). Data for the GBD database were derived from vital registries, verbal autopsies, censuses, household surveys, disease-specific registries, health service contact data, and other sources, ranging from 2.5% to 97.5% coverage, generating 95% upper intervals (UI) for all final estimates (13). This information, accessible at https://vizhub.healthdata.org/gbd-results, was overseen by the Institute for Health Metrics and Evaluation at the University of Washington in the USA (17). Joint point regression models for incidence, prevalence, and DALY rates in China and G20 countries. Ethical approval was not required as no human subjects were directly involved.

### 2.3 Statistical analyses

Age-standardized prevalence and death rates (ASPR, ASDR) for HHD in China and G20 countries are presented with 95% uncertainty intervals (UIs), stratified by age and sex. All metrics are expressed per 100,000 population. Statistical significance for all tests was defined by a two-sided *p*-value of less than 0.05.

The temporal distribution characteristics of the HHD burden were analyzed using a linked-point regression model composed of a series of statistical linear models. The age-standardized rate (ASR) is calculated using the direct standardization method. The standard population age structure weights employed originate from the “World Standard Population” defined by the GBD 2021 study. This method aims to eliminate differences in age composition between comparison populations, ensuring the comparability of rate values. In the GBD 2021 study, the ASR was calculated using the following formula: $\mathrm{ASR}=\frac{\sum_{i=1}^{A}\;a_{i}w_{i}}{\sum_{i=1}^{A}\;w_{i}}\times 100,000$, where $a_{i}$ represents the *i*th age group and the number of populations (or weight $w_{i}$) (15).

The 30-year trends from 1990 to 2021 for prevalence, mortality, and disability-adjusted life years (DALYs) were analyzed by calculating the average annual percent change (AAPC) and its 95% confidence intervals (CIs) based on age-standardized rates (ASRs). The AAPC was derived through a sequential log-linear regression procedure. This process began by fitting the natural logarithm of the ASRs against time as the independent variable to obtain the regression coefficient (*β*₁). Subsequently, this coefficient was converted into the final AAPC value through application of the standard formula: $\mathrm{AAPC}=(e^{\beta_{1}}-1)\times 100\text{\%}$ (18).

Joinpoint regression analysis (Joinpoint 5.3.0, NCI) was employed to characterize temporal trend patterns in HHD, with significant change points detected using Monte Carlo permutation tests. The model computed both segment-specific annual percentage change (APC) and the overall average annual percentage change (AAPC) for the period 1990–2021. A *p*-value less than 0.05 indicated that the APC and AAPC were statistically significantly different from zero, with the direction of the estimate (positive or negative) reflecting increasing or decreasing trends in the age-standardized rates, respectively.

We employed the ARIMA (p, d, q) framework to project HHD prevalence and mortality rates (ASPR, ASDR) in China by sex from 2021 to 2051. The model parameters represent the autoregressive order (*p*), the degree of differencing (d), and the moving average order (q). The modeling process comprised four key stages: (1) Stationarity Testing: The ADF test was applied to verify series stationarity. (2) Model Identification: Initial *p* and q orders were inferred from ACF and PACF plots. (3) Model Selection: The auto.arima() function was used to select the best-optimized model based on the Akaike information criterion (AIC). (4) Diagnostic Checking: The Ljung–Box Q test and residual ACF/PACF analyses confirmed the white noise properties of the residuals. Following successful diagnostics, the model was employed for forecasting. All statistical computations and graphics were generated using R 4.4.3 with the “forecast”, “series”, and “ggplot2” packages, adopting a significance threshold of *P* < 0.05 (19). The predicted starting point is derived by backward calculation from the differentiated sequence, which may exhibit slight deviations from the final observed value.

## 3 Results

Table 1 compares the burden of disease between China and G20 countries from 1990 to 2021, presenting case numbers, prevalence rates, mortality counts, ASPR, ASDR, DALYs, and AAPC. The associated 95% uncertainty intervals (UIs) are provided in parentheses.

**Table 1:** Comparison of all-age and age-standardized metrics for the prevalence, mortality, and DALYs of HHD between China and G20 countries in 1990 and 2021, with average annual percent change (AAPC).

Location	Measure	1990		2021		
		All-ages cases	Age-standardized rate per 100,000	All-ages cases	Age-standardized rate per 100,000	1990–2021 AAPC
		*n* (95% UI)	*n* (95% UI)	*n* (95% UI)	*n* (95% UI)	*n* (95% CI)
China	Prevalence	1,503,019 (1,161,178, 1,916,382)	218.24 (169.31, 274.83)	3,912,158 (2,989,417, 5,056,002)	192.47 (146.68, 245.02)	−0.4409 (−0.5412, −0.3404)
	Deaths	232,479 (155,806, 275,584)	42.64 (30.32, 49.56)	328,119 (224,717, 425,288)	18.85 (12.89, 24.47)	−2.6130 (−2.8397, −2.3858)
	DALYs	4,971,332 (3,273,139, 5,916,486)	716.02 (488.69, 841.36)	5,589,287 (3,973,271, 7,160,215)	292.54 (208.19, 374.2)	−2.9016 (−3.0832, −2.7196)
G20	Prevalence	3,311,734 (2,600,158, 4,193,117)	116.07 (90.82, 146.09)	9,081,974 (7,052,601, 11,561,802)	139.64 (109.06, 177.32)	0.5822 (0.5333, 0.6312)
	Deaths	485,563 (394,601, 538,978)	18.41 (15, 20.41)	896,718 (754,505, 1,011,003)	14.06 (11.83, 15.86)	−0.8754 (−1.0633, −0.6870)
	DALYs	10,232,241 (8,177,137, 11,419,351)	351.07 (284.34, 390.57)	16,018,851 (13,697,747, 18,014,322)	248.75 (212.81, 279.27)	−1.0945 (−1.1986, −0.9903)

G20, group of 20; UI, uncertainty interval; CI, confidence interval; DALYs, disability-adjusted life years; AAPC, average annual percentage change.

### 3.1 Number of cases, incidence rate, number of deaths, mortality rate, number of DALYs, and DALY rate by age and gender in 2021

**Figure 1:**
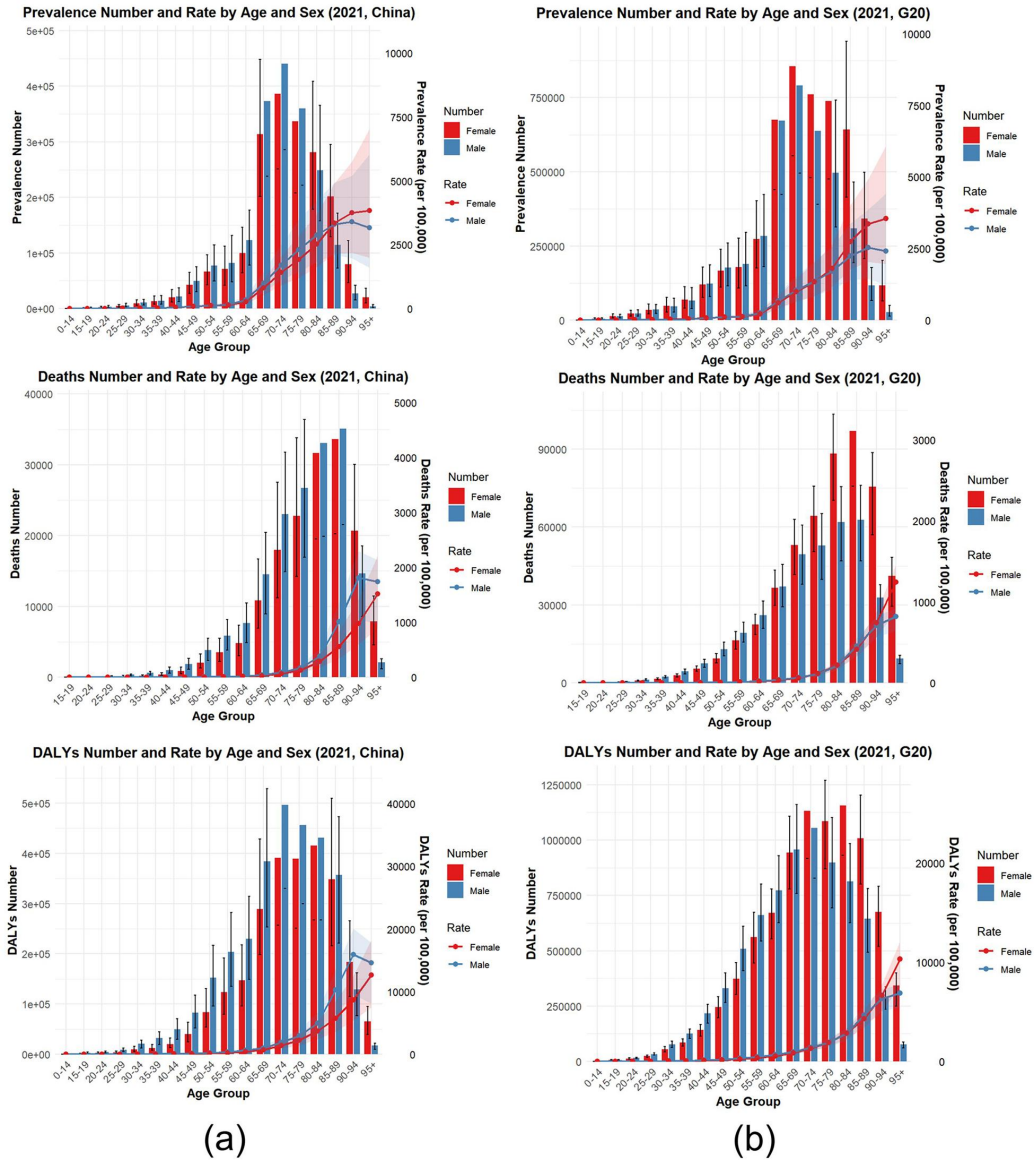
This figure shows the age- and sex-specific burden of hypertensive heart disease (HHD) in China **(a)** and G20 countries **(b)** in 2021, encompassing prevalent cases, prevalence, death counts, mortality rates, DALY counts, and DALY rates. The shaded areas indicate the 95% uncertainty intervals (UI), demonstrating the overlap between male and female data.

As shown in Figure 1, in 2021, the number of HDD cases, deaths, and DALYs in China and G20 countries were primarily concentrated in the 65–69, 70–74, and 75–79 age groups. The peak number of cases in both China and G20 countries occurred in the 70–74 age group. Within this group, China recorded 8.27 million cases (95% UI: 5.37–12.04 million), while G20 countries reported 16.47 million cases (95% UI: 10.52–24.06 million). The peak number of deaths in China and G20 countries occurred in the 75–79 age group. Within this group, China recorded 0.41 million deaths (95% UI: 0.28–0.53 million), while G20 countries recorded 1.02 million deaths (95% UI: 0.85–1.16 million). The peak number of DALY cases in China and G20 countries occurred in the 70–74 age group. Within this group, China recorded 8.87 million cases (95% UI: 6.38–11.38 million), while G20 countries recorded 21.88 million cases (95% UI: 18.58–24.87 million). In 2021, the prevalence, mortality, and DALY rates for HHD in China and G20 countries will increase with age, approaching zero before the 60–64 age group. Prevalence shows an annual upward trend after age 65, while mortality and DALY rates exhibit a rapid increase and peak after the 75–79 age group. No significant differences in prevalence, mortality, or DALY rates were observed between genders across all age groups. These findings indicate that in 2021, the disease burden of HHD remained high in both China and G20 countries. The prevalence, mortality, and DALY rates of HHD in China and G20 countries increase with age, suggesting that the disease burden is primarily concentrated among the elderly population. Given the current stage of global aging, preventing and controlling HHD is particularly crucial. Notably, China's HHD cases, deaths, and DALYs each represent approximately half of the G20 average. The increase in DALYs is largely attributable to population growth and aging (13).

### 3.2 Hypertensive heart disease: A comparative study of China and G20 countries (1990–2021)

#### 3.2.1 ASPR

**Figure 2:**
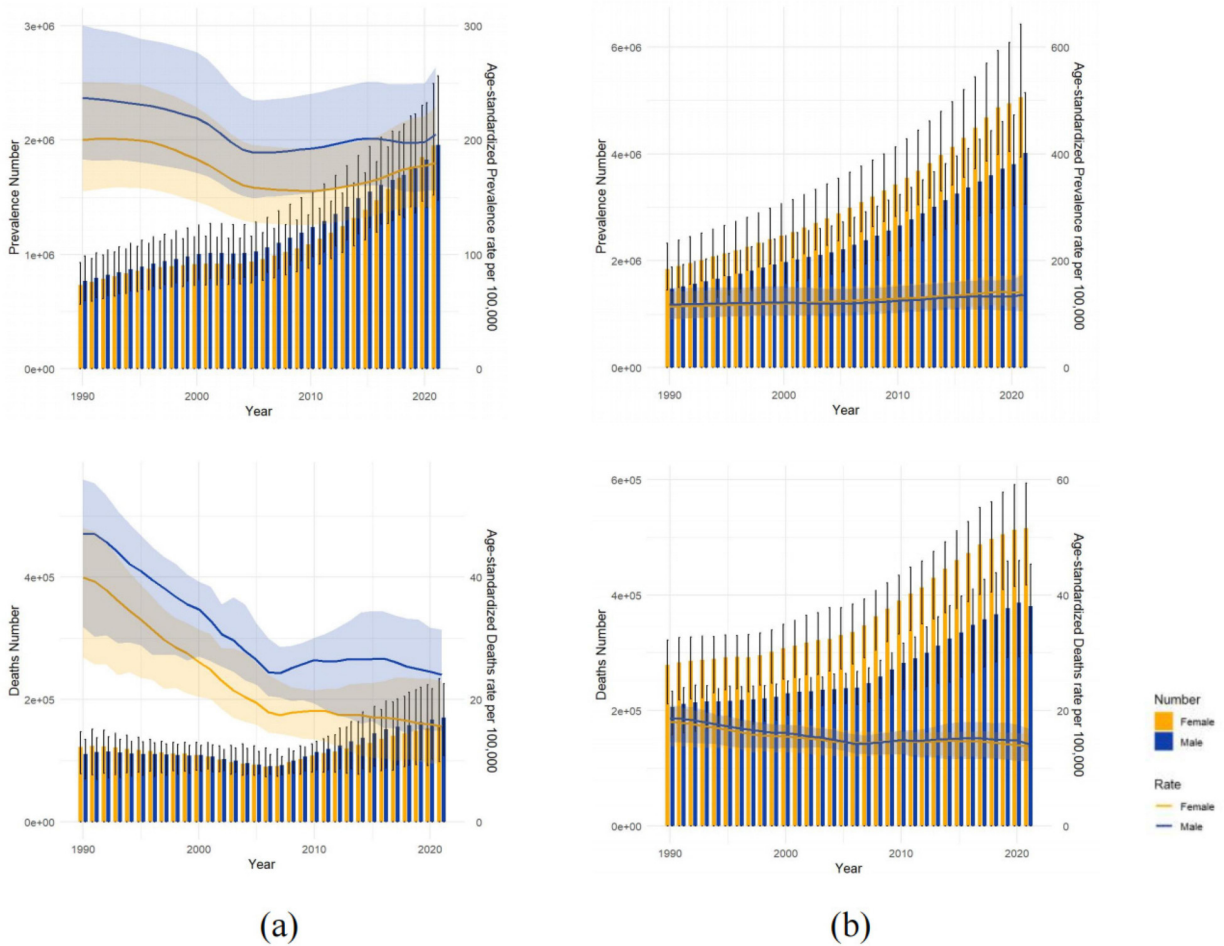
This figure represents the number of prevalent cases, ASPR, number of death cases, and ASDR for males and females in China **(a)** and the G20 countries **(b)** from 1990 to 2021. The blue and yellow shaded areas indicate 95% UI, and the grey shaded areas indicate overlap between them.

From 1990 to 2021, China's male HHD ASPR remained consistently higher than the female rate, with both showing a declining trend (Figure 2). Specifically, the male ASPR decreased from 237 per 100,000 population to 205 per 100,000 population, while the female ASPR declined from 200 per 100,000 population to 180 per 100,000 population. In G20 countries, no significant gender disparity was observed in HHD ASPR. Overall, male HHD ASPR was slightly higher than female ASPR, with both showing a slight upward trend. Male ASPR increased from 117 per 100,000 population to 137 per 100,000 population. The female ASPR increased from 113 per 100,000 population to 140 per 100,000 population. Comparatively, China's HHD ASPR was higher overall than that of G20 countries, but China's HHD ASPR showed a declining trend, whereas G20 countries showed a declining trend. Number of cases: China: The number of cases increased from 15.03 million (11.61–19.16) to 39.12 million (29.89–50.56) (+106.28%). G20 countries: The number of cases increased from 33.11 million (26–41.93) to 90.81 million (70.52–115.61) (+174.26%).

#### 3.2.2 Deaths cases and ASDR

In China, the Age-Standardized Death Rate (ASDR) for HHD decreased from 42 to 18 per 100,000. However, the annual number of deaths increased by 41.3%, from 2.32 million (95% UI, 1.55–2.75) to 3.28 million (95% UI, 2.24–4.25). Similarly, the Age-Standardized DALY Rate fell from 716 to 292 per 100,000, while the number of DALY cases rose by 12.43%, from 49.71 million (95% UI, 32.73–59.16) to 55.89 million (95% UI, 39.73–71.6).Among G20 countries, the ASDR declined from 18 to 14 per 100,000. Conversely, the annual death count saw a more substantial increase of 84.74%, from 4.85 million (95% UI, 3.94–5.38) to 8.96 million (95% UI, 7.54–10.11).

### 3.3 Joinpoint regression analysis

**Figure 3:**
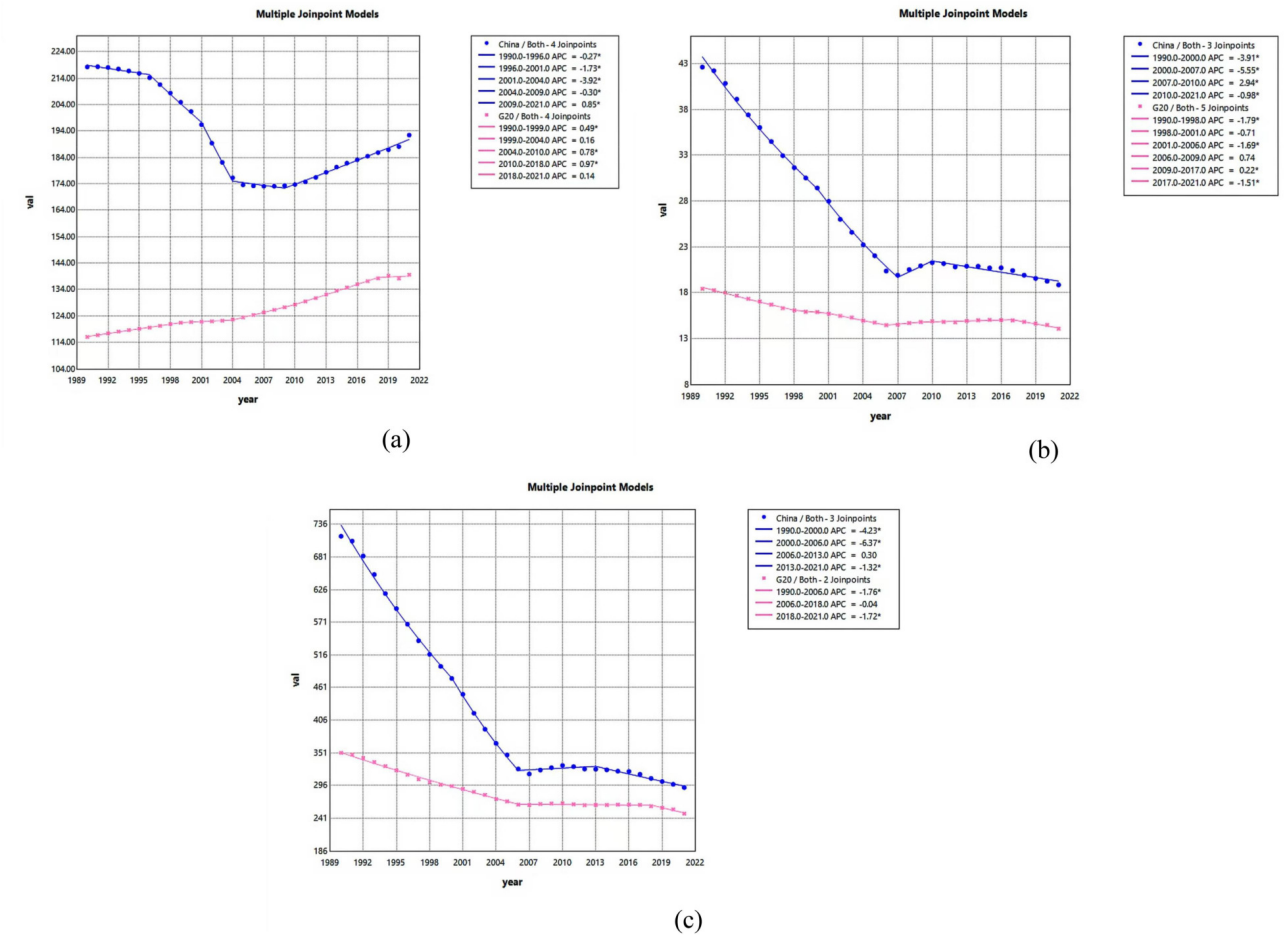
This figure shows the APC of the ASPR, ASDR, and DALYs for HHD in China and G20 countries from 1990 to 2021 (* indicates a *p*-value <0.05, which is significantly different from 0); APC <0 shows a decreasing trend, and APC >0 shows an increasing trend. Plots for ASPR **(a)**, ASDR **(b)**, and DALY **(c)** for China and G20 countries, with data from China highlighted in blue and data from G20 countries in red.

As shown in Figure 3, from 1990 to 2021, China's ASPR, ASDR, and DALYs for HHD exhibited an overall downward trend, with AAPCs of −0.4409% (95% UI = −0.5412, −0.3404), −2.6130% (−2.8397, −2.3858), and −2.9016% (−3.0832, −2.7196) (Table 1). APCs for ASPR among Chinese HHD patients showed a consistent downward trend from 1990 to 2009, with a significant decline observed between 2001 and 2004 (APC = −3.92%, significantly different from zero). Conversely, ASPR exhibited an upward trend from 2001 to 2009 (APC = 0.85%). APCs for ASDR among Chinese HHD patients showed declining trends in 1990–2000, 2000–2007, and 2010–2010, with APCs of −3.91%, −5.55%, and −0.98%, respectively. Conversely, a rising trend was observed in 2007–2010, with an APC of 2.94%. APCs for DALYs among Chinese HHD patients showed a decreasing trend in 1990–2000, 2000–2006, and 2013–2010, with rates of −4.23%, −6.37%, and −1.32%, respectively. However, a slight increase was observed in 2006–2013, with an APC of 0.03% (Figure 3).

APCs for ASPR among G20 HHD patients showed an overall upward trend from 1990 to 2021. When divided into five five-year intervals, APCs fluctuated between 0.14% and 0.97%, showing significant differences from zero. China's APCs for ASDR in HHD patients showed declining trends in 1990–1998, 1998–2001, 2001–2006, and 2017–2021, at −1.79%, −0.71%, −1.69%, and −1.51%, respectively. Conversely, APCs increased during 2006–2009 and 2009–2017, with values of 0.74% and 0.22%. APCs for DALYs among G20 countries' HHD patients showed an overall upward trend from 1990 to 2021. Dividing this period into three intervals yielded values of −1.76%, −0.04%, and −1.72%, respectively, all significantly different from zero (Figure 3).

### 3.4 Projected trends in HHD prevalence and mortality in China (2021–2051)

**Figure 4:**
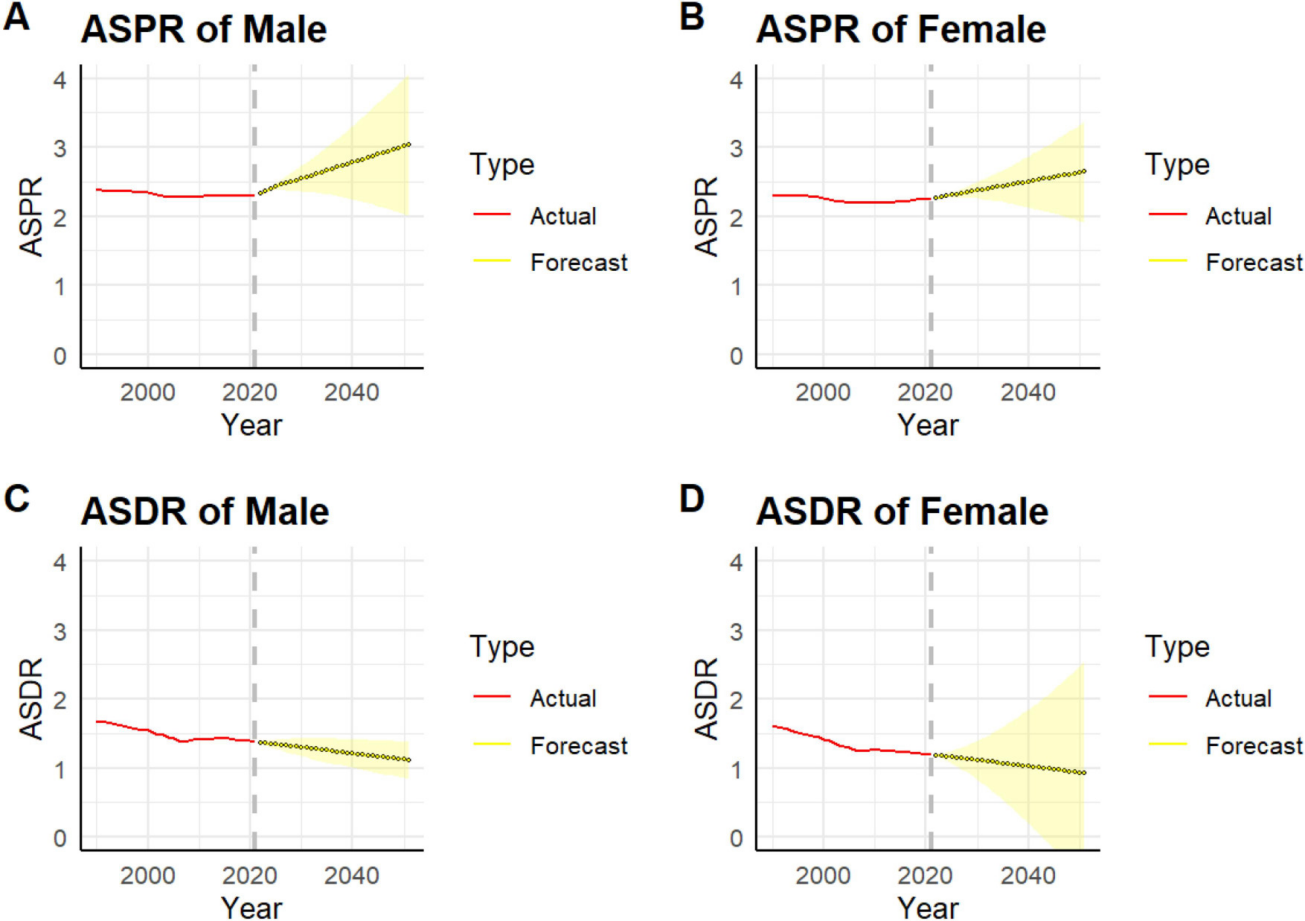
This figure shows the actual and predicted prevalence rates of HHD for men **(a)** and women **(b)** in China from 1990 to 2021 and up to 2051, along with the actual and predicted death rates of HHD for men **(c)** and women **(d)** the red solid line represents the actual value, the yellow dashed line represents the predicted value, and the yellow shaded area represents the 95% UI.

As shown in Figure 4, After filtering via the auto.arima() function, the optimal model for male prevalence was determined to be ARIMA(2,2,0) (AIC = −279.59),we find that the prevalence rate among males is projected to rise significantly from 2.31% in 2021 to 3.03% in 2051, reflecting an increase of 0.72 percentage points (relative increase: 31.1%). The prevalence rate for females is projected to increase significantly from 2.25% in 2021 to 2.64% in 2051, reflecting a rise of 0.39 percentage points (relative increase: 17.3%) [ARIMA(3,2,0), AIC = −292.83]. The mortality rate for males is projected to decrease from 1.38% in 2021 to 1.11% in 2051, reflecting a decline of 0.39 percentage points (relative decrease: 35.1%) [ARIMA (1,1,0), AIC = −199.46]. The mortality rate for women is projected to decrease from 1.19% in 2021 to 0.92% in 2051, reflecting a decline of 0.27% (relative decrease: 22.7%) [ARIMA (0,2,0), AIC = −199.32]. ARIMA model projections indicate that by 2051, the age-standardized prevalence of HHD will enter a plateau phase of slow decline [Men: ARIMA (2,2,0), MAPE = 5.5%; Females: ARIMA (3,2,0), MAPE = 5.2%]. Notably, gender difference analysis indicates that the disease burden remains consistently higher among males than females, yet mortality improvement exhibits distinct gender patterns (Figure 4).

## 4 Discussion

This study systematically assessed the disease burden of hypertensive heart disease in China and G20 countries from 1990 to 2021 and projected trends for China over the next 30 years. First, despite China's significant achievements in reducing HHD's age-standardized prevalence rate (ASPR), age-standardized mortality rate (ASDR), and disability-adjusted years (DALYs) over the past 30 years, China's current ASIR and ASMR for HHD remain higher than the G20 average. Furthermore, the number of HHD cases and deaths in China has increased substantially, with the majority of patients being elderly. This phenomenon likely stems from China's substantial investments in HHD prevention and control over recent decades. However, China continues to face challenges posed by its large population base and severe aging trends. The decline in China's age-standardized HHD burden metrics likely stems from substantial investments over recent decades in widespread hypertension screening, health lifestyle guidance, and broad access to effective antihypertensive medications. Research indicates that combining physical activity with healthy dietary patterns significantly reduces hypertension risk, underscoring the importance of lifestyle interventions in prevention. Increasing physical activity and adopting healthy dietary patterns effectively lower hypertension risk; replacing 21 min of sedentary time with running or stair climbing significantly improves blood pressure (20, 21). However, the substantial rise in both morbidity and mortality cases highlights two key factors. On one hand, this likely reflects the profound impact of population aging. This means that even with controlled relative disease risks, the sheer size of the elderly population will sustain a heavy disease burden, placing continuous pressure on healthcare systems. On the other hand, advances in medical technology and heightened public health awareness have led to the diagnosis of milder symptoms, statistically contributing to the apparent increase in prevalence (22). It is evident that the burden of HHD in China remains significant, and preventing and controlling HHD continues to be crucial for ensuring a high-quality life for the people.

Second, China and G20 nations exhibit starkly divergent historical trajectories: China's burden metrics have generally declined, while G20 countries show overall stability or slight increases. During this period, China achieved significant reductions in age-standardized prevalence rates (ASPR), age-standardized mortality rates (ASDR), and disability-adjusted life years (DALY) rates, with average annual percentage changes (AAPC) of −0.44%, −2.61%, and −2.90%, respectively. This positive trend likely stems from China's sustained public health investments over the past three decades. These include nationwide hypertension screening programs, large-scale public health education promoting low-salt diets and active lifestyles, and expanded coverage and accessibility of essential antihypertensive drugs within the national medical insurance catalog (7, 23). Notably, the new round of healthcare system reforms initiated in 2009 reinforced the role of primary healthcare services in chronic disease management, profoundly impacting standardized hypertension management and thereby effectively controlling HHD (24). In contrast, the age-standardized prevalence rate (ASPR) of HHD across G20 nations, including China, showed a slight yet statistically significant upward trend (AAPC = 0.34%). This indicates that the disease burden of HHD remains unchecked in major global economies.Potential reasons are multifaceted. First, many G20 member states are experiencing a persistent rise in the prevalence of metabolic risk factors such as obesity and diabetes, which are key drivers of HHD (25). Second, despite advances in medical technology, awareness, treatment, and control rates for hypertension remain at an unsatisfactory plateau in some countries, particularly low- and middle-income members (26). Moreover, the global trend of population aging represents an underlying factor that cannot be overlooked. The absolute increase in the elderly population directly contributes to the accumulation of HHD cases. Regarding gender disparities, males consistently bear a higher disease burden, potentially linked to their greater exposure to behavioral risk factors such as smoking and alcohol consumption (27).

Joinpoint regression analysis further refines our understanding of historical trends. The brief upward inflection point in China's age-standardized mortality rate (ASMR) between 2007 and 2010 may be linked to improvements in medical diagnostics, enhanced cause-of-death registration systems, or shifts in risk factors among specific subgroups during this period, warranting further investigation. First, China's new round of healthcare system reforms launched in 2009 significantly enhanced primary care capabilities and cardiovascular disease diagnosis awareness, potentially leading to more accurate reporting of previously underrecognized HHD-related deaths (24). Second, the continuous refinement of China's national cause-of-death surveillance system during this period, with improvements in reporting quality and coverage, may inherently elevate reported mortality rates (28). This inflection point underscores the importance of distinguishing genuine epidemiological shifts from the impact of surveillance system advancements when interpreting long-term disease burden trends. The long-term, gradual rise in ASPR among G20 nations serves as a warning: globally, the prevalence of HHD risk factors—such as obesity, high-sodium diets, and sedentary lifestyles—may be offsetting or even reversing the benefits gained from medical advancements. This trend suggests that the fundamental solution to HHD lies in moving beyond clinical treatment alone to prioritize preventive strategies that integrate population-level lifestyle interventions with clinical care.

Finally, projections for the next three decades reveal a daunting challenge: China's HHD prevalence is expected to continue rising. Although mortality rates are projected to decline further, China will still face a substantial HHD disease burden over the next 30 years. The ARIMA model predicts that China's HHD prevalence will rise while mortality declines, revealing a core contradiction: advances in medical technology can effectively delay patient death (reducing ASDR) but struggle to reverse disease progression and incidence (leading to cumulative increases in ASPR). First, the cumulative rise in ASPR may stem from increased public health awareness and higher screening rates. The ease of home blood pressure monitoring has boosted diagnosis rates. Additionally, in the current digital era, rising prevalence of depression and anxiety may contribute to the rising HHD prevalence (29, 30). Moreover, while hypertension prevalence is rising in China, awareness, treatment, and control rates remain relatively low. Inadequate blood pressure management and reinforcement can damage cardiac structure and function, ultimately leading to hypertensive heart disease (23, 26). With population aging, this trend signals that China will face an increasingly large HHD patient population in the future, likely linked to population aging trends. It is estimated that by 2030, over one billion people will be classified as elderly, defined as those aged 65 and above (31). China's aging population is progressively intensifying; according to the Seventh National Population Census, the proportion of individuals aged 60 and above has already exceeded 18% (2). This will place greater strain on healthcare systems including increased demand for medical resources and the need to enhance the quality and accessibility of healthcare services. This study has several limitations. First, GBD data are inherently model-based estimates; despite rigorous methodology, they may be influenced by the quality of source data and modeling assumptions. Second, although the GBD methodology strives for standardization, differences among G20 countries in hypertension diagnostic criteria, cause-of-death coding practices, and the sophistication of health information systems may affect the precision of cross-national comparisons. Third, ARIMA forecasting primarily relies on inertial extrapolation of historical data, failing to anticipate potential future breakthroughs in technology or robust public health policy interventions. Fourth, this study primarily analyzes data at the national level, failing to reveal disparities in disease burden between urban and rural areas or across regions within China. Future research should conduct in-depth subnational analyses to guide more precise resource allocation. Additionally, as a member of the G20, China's data is included in the overall G20 statistics, which to some extent affects the independence of inter group comparisons. Future research could conduct in-depth comparative analyses between China and other G20 countries.

## 5 Conclusion

In summary, this study systematically assessed the disease burden of hypertensive heart disease (HHD) in China and the G20 as a whole by analyzing data from 1990 to 2021 and projecting future trends in China. Key findings indicate that despite China's significant achievements in reducing age-standardized prevalence rates (ASPR) and age-standardized mortality rates (ASDR) for HHD, the aging population has driven a continuous rise in the number of affected individuals. The current burden level remains higher than the G20 average. Unlike the slight upward trend in G20 prevalence rates, China exhibits a declining trajectory, the timing coincides with concurrent national public health initiatives such as large-scale hypertension screening, primary care reinforcement, and improved drug accessibility, suggesting that these combined measures may have produced positive effects. Projections indicate that China's HHD prevalence will continue to rise over the next 30 years, signaling that a large and growing population of chronic disease patients will place long-term pressure on the healthcare system. To address this formidable challenge, China must implement tailored integrated strategies. While advancing primary prevention of hypertension and strengthening risk factor control, it is imperative to significantly enhance primary healthcare systems and establish patient-centered, comprehensive management models for HHD chronic diseases. This approach will more effectively reduce the disease burden and improve population health levels (8).

## Data Availability

The original contributions presented in the study are included in the article/Supplementary Material, further inquiries can be directed to the corresponding author.
